# Continuous beta-lactam infusion in critically ill patients: the clinical evidence

**DOI:** 10.1186/2110-5820-2-37

**Published:** 2012-08-16

**Authors:** Mohd H Abdul-Aziz, Joel M Dulhunty, Rinaldo Bellomo, Jeffrey Lipman, Jason A Roberts

**Affiliations:** 1 Burns, Trauma and Critical Care Research Centre, University of Queensland, Brisbane, Australia; 2 Department of Intensive Care Medicine, Royal Brisbane and Woman’s Hospital, Brisbane, Australia; 3 Department of Intensive Care, Austin Hospital, Melbourne, Australia; 4 Pharmacy Department, Royal Brisbane and Woman’s Hospital, Brisbane, Australia

**Keywords:** Beta-lactam antibiotic, Continuous infusion, Critically ill, Pharmacokinetic, Pharmacodynamic, Treatment outcome

## Abstract

There is controversy over whether traditional intermittent bolus dosing or continuous infusion of beta-lactam antibiotics is preferable in critically ill patients. No significant difference between these two dosing strategies in terms of patient outcomes has been shown yet. This is despite compelling in vitro and in vivo pharmacokinetic/pharmacodynamic (PK/PD) data. A lack of significance in clinical outcome studies may be due to several methodological flaws potentially masking the benefits of continuous infusion observed in preclinical studies. In this review, we explore the methodological shortcomings of the published clinical studies and describe the criteria that should be considered for performing a definitive clinical trial. We found that most trials utilized inconsistent antibiotic doses and recruited only small numbers of heterogeneous patient groups. The results of these trials suggest that continuous infusion of beta-lactam antibiotics may have variable efficacy in different patient groups. Patients who may benefit from continuous infusion are critically ill patients with a high level of illness severity. Thus, future trials should test the potential clinical advantages of continuous infusion in this patient population. To further ascertain whether benefits of continuous infusion in critically ill patients do exist, a large-scale, prospective, multinational trial with a robust design is required.

## Review

### Introduction

The mortality rate of severe sepsis and septic shock in critically ill patients remains high despite recent therapeutic advances. Swift and judicious antibiotic use in these patients is vital and any delays are associated with increases in mortality [1,2]. Beta-lactam antibiotics are used commonly and are regarded as a cornerstone in the management of critically ill patients with severe sepsis in intensive care units (ICU) around the world [1,3,4]. However, the occurrence of severe pathophysiological changes, namely the fluid shift phenomenon [5] and augmented renal clearance [6], in critically ill patients may alter the pharmacokinetics (PK) of the antibiotics. Thus, appropriate dosing modifications should be applied to prevent inadequate antibiotic concentrations and therapeutic failure [5,7,8].

**Figure 1:**
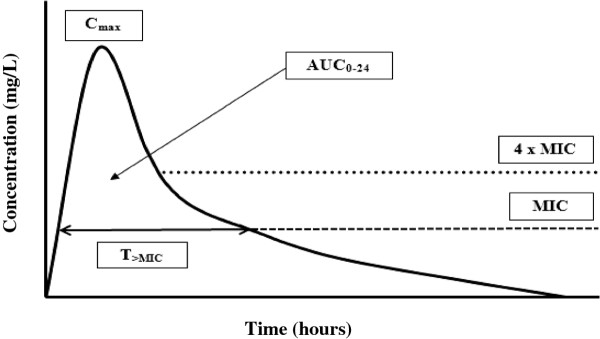
**Pharmacokinetic and pharmacodynamic parameters of antibiotics on a concentration vs. time curve.** T_>MIC_, time that a drug’s plasma concentration remains above the minimum inhibitory concentration (MIC) for a dosing period; C_max_, maximum plasma antibiotic concentration; AUC_0-24_, area under the concentration-time curve during a 24-hour time period.

Antibiotic pharmacodynamics (PD) is the discipline that attempts to relate PK parameters to the ability of an antibiotic to kill or inhibit the growth of bacterial pathogens [9]. Antibiotics can be classified based on these PD characteristics. Generally, antibiotics are classified into three categories based on their mode of bacterial killing: (1) concentration-dependent; 2) time-dependent; or 3) both (Figure 1). The first category includes antibiotics, such as aminoglycosides, where the best predictor of efficacy is the ratio of peak drug concentration (C_max_) to minimum inhibitory concentration (MIC; C_max_/MIC) [10,11]. Some antibiotics, such as fluoroquinolones and glycopeptides, are more complex and exhibit both a concentration and time-dependent kill characteristics where the best predictor of efficacy is the ratio of area under the concentration time curve during a 24-hour period (AUC_0-24_) to MIC (AUC_0-24_/MIC). Therefore, increasing the dose or/and concentration for these antibiotics can be logically expected to enhance the rate and extent of bacterial killing [12,13]. In contrast, higher beta-lactam concentrations do not significantly influence their efficacy. Based on numerous in vitro and in vivo experimental data, it is the duration of effective antibiotic exposure that is more important for these time-dependent antibiotics [14-17].

**Figure 2:**
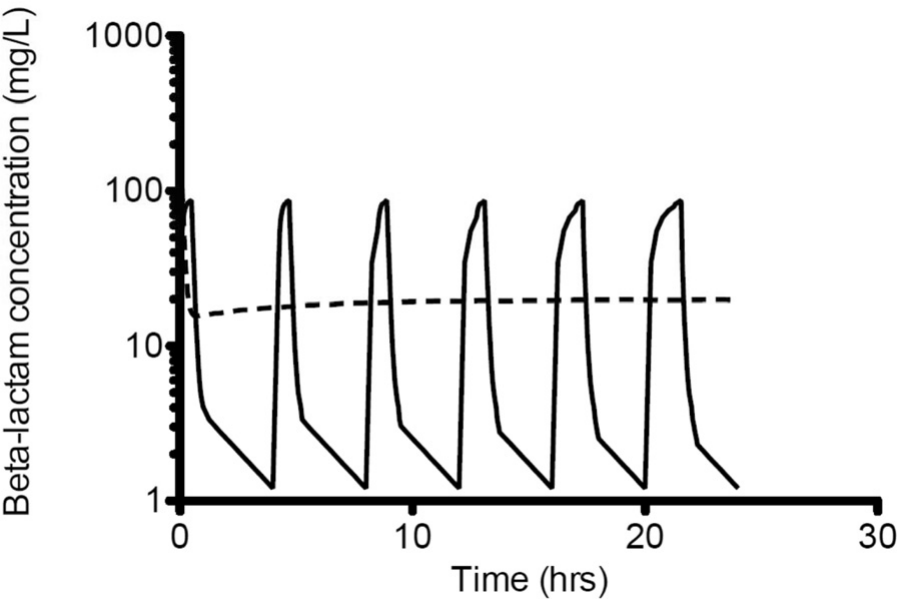
**The simulated concentration-time profile of a beta-lactam antibiotic when administered by intermittent bolus dosing or continuous infusion (Vd = 0.22 L/kg; T**_**1/2**_ **= 2.45 hr).** Intermittent bolus dosing (solid lines); continuous infusion (dotted lines).

**Figure 3:**
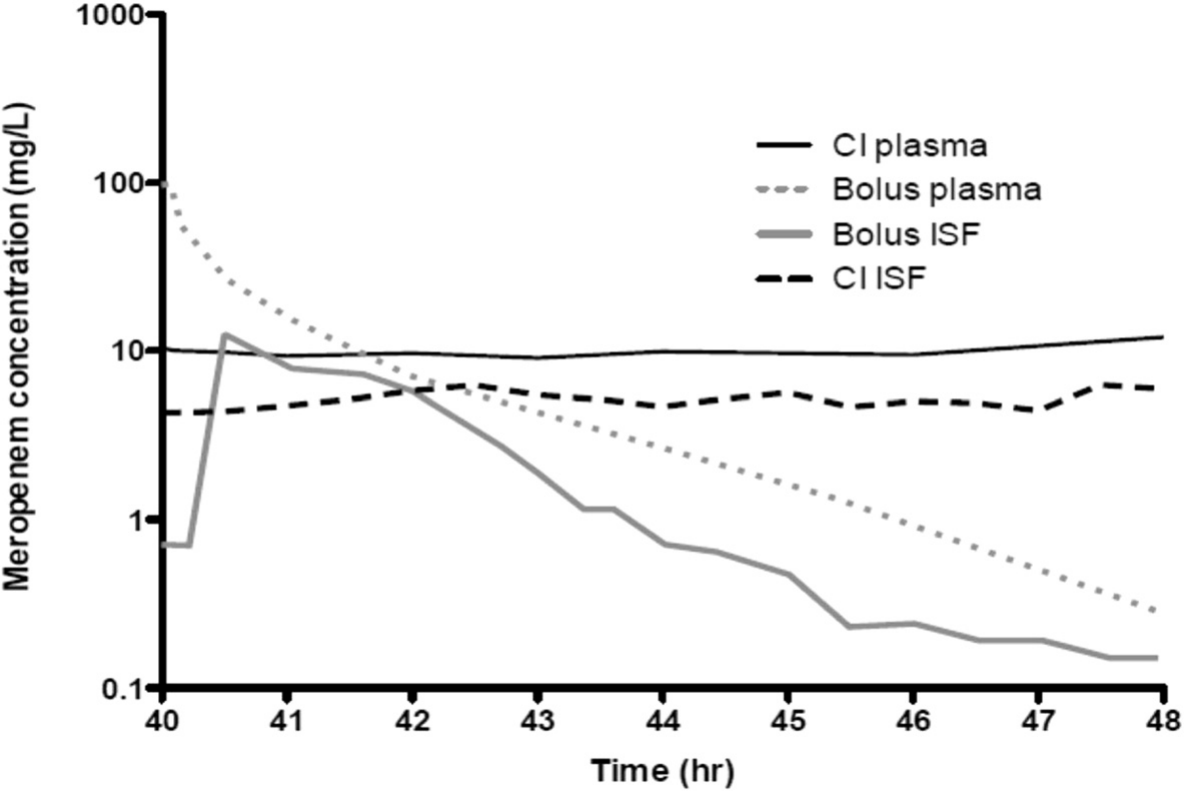
**Observed steady-state plasma and tissue concentrations for meropenem administered to critically ill patients with sepsis by intermittent bolus dosing and continuous infusion (adapted from Roberts et al. 2009, J Antimicrob Chemother).** Continuous infusion (CI) meropenem plasma concentration (solid dark lines); IB meropenem plasma concentration (dotted grey lines); IB meropenem interstitial fluid concentration (ISF) (solid grey lines); CI meropenem ISF concentration (dotted dark lines).

The debate persists about whether traditional intermittent bolus dosing (IB) or continuous infusion (CI) is clinically preferable for administration of beta-lactam antibiotics. This is despite the fact that beta-lactam PD data suggest advantages for CI compared with IB [18-24], showing time-dependent activity and demonstrating that the duration of time (T) the free drug concentration remains above the minimum inhibitory concentration (MIC; *f*T_>MIC_) best describes its bacterial kill characteristics [15] (Figure 1). Thus, administration via CI should be advantageous, because it inevitably produces higher and sustained antibiotic concentrations above the MIC. It also is noteworthy that IB yields unnecessary high peak and low trough concentrations below MIC for much of the dosing interval [25-28] (Figure 2 and Figure 3). The constant and sustainable antibiotic concentrations provided by CI are particularly important for pathogens with high MIC values. Such pathogens are relatively common in the ICU [29-31].

Despite these theoretical advantages, a global practice shift toward CI of beta-lactam antibiotics has not taken place. This is mostly because, although CI has been shown to be superior to IB dosing during in vitro [32,33] and in vivo [34-37] experimental studies, comparative clinical studies have so far failed to demonstrate significant differences in patient outcome. Furthermore, three recent meta-analyses of these clinical trials have found similar outcomes between CI and IB, in heterogeneous hospitalized patient populations [38-40]. This dissociation between preclinical data and clinical reports raises uncertainty for the treating clinician. Importantly, most trials have important methodological flaws and have used inconsistent methods and therapeutic endpoints [29]. There also is a lack of general consensus about which patient groups should be investigated and the appropriate methodology that should be employed to identify whether clinical outcome differences between these two dosing approaches exist in all hospitalized patients. The possible advantages and disadvantages from the two dosing methods are further summarized in Table 1.

**Table 1:** Possible advantages and disadvantages of employing continuous or intermittent administration of beta-lactam antibiotics

**Administration method**	**Advantages**	**Disadvantages**
Continuous infusion	More predictable antibiotic PK profiles	Relatively new antibiotic administration method thus requiring intensive educational effort to update clinical staff on the administration method prior to implementation
	Lower antibiotic daily dose may be appropriate with continuous infusion	Requires special infusion pumps and infusion bags that are costly
	Reduced drug acquisition costs when lower antibiotic doses are used	Some beta-lactams (e.g., meropenem) are not stable under prolonged exposure at room temperature and may produce and enhance degradation products that cause hypersensitivity reactions
	Effective resource consumption (e.g., reduce the time required for pharmacists or nurses to prepare and administer antibiotic)	Risk of drug wastage is high with this approach (e.g., when treatment ceased before infusion bag completed)
Intermittent bolus	Simple	PK/PD targets may not be achieved (especially in critically ill patients)
	Does not require dedicated line access for drug administration (incompatibility issues unlikely)	Neurological adverse effects are theoretically more possible with high C_max_
	Less likely to have unexpected device failures and dosing delivery rate errors	

The purpose of this review is to describe the published clinical trials and their associated methodological shortcomings in their comparison of CI and IB administration in hospitalized patients. Several intriguing issues or problems involved in the interpretation of results obtained from the available studies also will be highlighted. Finally, based on these discussions, description of a methodologically robust, definitive clinical trial will be proposed.

### PK/PD considerations

The percentage (%) of *f*T_>MIC_ (% *f*T_>MIC_) during a dosing interval is regarded as the optimal PD index for beta-lactam antibiotics, and as such, maintaining effective drug concentration above the MIC should be the priority when this antibiotic class is used. Specifically, the % *f*T_>MIC_ needed for bacteriostasis and bactericidal is 35-40% and 60-70% for cephalosporins, 30% and 50% for penicillins, 20% and 40% for carbapenems, respectively [9,33,41,42]. However, it is imperative to note that these indices should be regarded as the minimum PD endpoints and that they may not be adequate to treat severe infections and to prevent the development of antibiotic resistance [43]. Furthermore, emerging retrospective clinical data for critically ill patients suggest patient benefits with higher and longer antibiotic exposures than those described for the in vitro and in vivo experimental studies [44,45]. Thus, it has been suggested that maintaining concentrations above the MIC for 90-100% of the dosing interval is a rational PD endpoint to ensure that the above minimum targets are achieved [46]. Combining the above data, beta-lactams should be more effective when delivered continuously to achieve a level above the MIC continuously throughout treatment. Alternatively, prolonging the infusion time via extended-infusion (EI), also has been suggested to maximize the *f*T_>MIC_ for this antibiotic class without some of the CI-associated drawbacks outlined in Table 1[47,48]. Both CI and EI may be particularly advantageous in the treatment of severe infections.

### Inconsistent PD endpoints for comparison

Different PD endpoints have been used in published studies, which make comparison between CI and IB difficult. However, several reviews have suggested that prolonged antibiotic exposures will achieve better PD profile [7,49,50]. Apparent benefits with regards to maximum bacterial killing also were reported in several studies when antibiotic concentrations were maintained above the MIC for extended periods, ideally four to five times the MIC especially when less susceptible microorganisms were involved [33,44,51-55]. In combination with other PK/PD data, it is suggested that therapeutic targets for CI therapy should be a steady-state concentration (C_ss_) that is at least 4 x MIC [33]. Thus, future comparative PK/PD studies should evaluate the relative ability of IB to achieve a trough concentration (C_min_) greater than the 4 x MIC of the offending pathogen for 40–70% of a dosing interval and for CI, a C_ss_ greater than 4 x MIC to prevent biased comparisons. It follows that the real challenge for IB is to obtain a C_min_ greater than 4 x MIC in a severely ill patient who is infected with a pathogen with high MIC.

### The role of postantibiotic effect

Another consideration for optimizing antibiotic pharmacokinetic exposure is the postantibiotic effect (PAE), i.e., the suppression of bacterial growth even with antibiotic concentrations below the MIC [56]. Although all antibiotics demonstrate PAE against susceptible gram-positive pathogens (i.e., staphylococci and streptococci), only some antibiotics, such as the aminoglycosides and fluoroquinolones produce prolonged PAE for gram-negative pathogens [15]. In contrast, beta-lactams except for the carbapenems, produce minimal or no PAE against gram-negative pathogens. It follows that the reduced % *f*T_>MIC_ required for carbapenems bacteriostatic (20%) and bactericidal (40%) activity may relate to the antibiotics PAE [56-61]. Therefore, the need for frequent dosing and continuous administration is deemed supplemental when antibiotics such as carbapenems display significant PAE.

### Revision in antibiotic breakpoints

The recent revision in antibiotic breakpoints to indicate if an organism is susceptible or resistant to different antibiotics, as classified by the European Committee on Antimicrobial Susceptibility Testing (EUCAST; available at http://www.eucast.org/clinical_breakpoints/) and Clinical and Laboratory Standards Institute (CLSI; available at http://www.clsi.org/) have had an impact on how clinicians view and manage infections worldwide. These new rules also are applicable for interpretation of antibiotic PK/PD studies. Due to these changes, future studies need to be interpreted in light of the new susceptibility breakpoints [62]. These new rules may mean that the present dosing approaches are more or less likely to achieve PK/PD targets [29].

### The role of optimal PK/PD targets in the prevention of antibiotic resistance

Antibiotic resistance patterns have significantly changed during the past 15 years with increasing resistance currently regarded as a major health crisis [63,64]. Furthermore, the rate at which the pathogens are currently developing antibiotic resistance is likely to far outpace the rate of development of new antibiotics. Thus, optimizing the use of new or existing antibiotics via PK/PD principles may prolong their lifespan in clinical practice [42,57]. Although numerous studies have been performed to determine the optimal PK/PD targets for clinical and bacteriological success, very little data exist that describe their roles in the prevention of bacterial resistance. However, extensive research in this area has been conducted with fluoroquinolones and more importantly, the corresponding optimal PD targets (i.e., AUC/MIC breakpoints) for resistance prevention has been described for this antibiotic class [65-67]. The success with fluoroquinolones further emphasizes that PK/PD principles are not only relevant in bacterial eradication but also should be considered to minimize the development of bacterial resistance. For the beta-lactams, however, limited data are currently available, with the exception of several in vitro [68,69] and in vivo experimental studies [70], which suggest the optimal PD targets for the prevention of resistance. Thus, appropriate targets are initially needed for this antibiotic class before a dosing regimen that minimizes resistance development can be recommended. Until convincing evidence becomes available, antibiotic dosing that targets concentrations greater than 4–6 x MIC for an extended interval should be aimed at to prevent resistance [33,71,72]. It also follows that once the targets are defined, it will then be possible to evaluate the relative ability of CI vs. IB in reducing the emergence of resistance associated with the use of beta-lactam antibiotics. However, several reviews have suggested that the currently proposed PK/PD target is probably best achieved by using CI or EI [29,46].

### Controversies surrounding data interpretation

**Figure 4:**
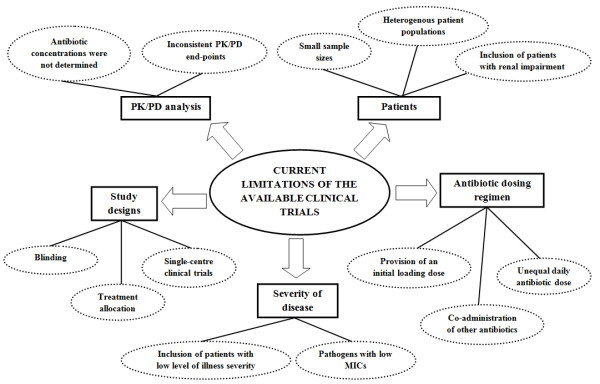
The summary of the current limitations and flaws associated with the available clinical trial.

Since the initial availability of antibiotics, methods to optimize antibiotic dosing have been explored [17,73]. Numerous trials have been conducted with beta-lactams testing various dosing strategies in various patient populations [52,74-89]. The characteristics and findings of these relevant clinical trials are described in Tables 1 and 2, respectively. However, these studies have not defined whether altered dosing approaches are advantageous and which patient groups may benefit. Most of these trials were conducted in North America and Europe between 1979 and 2008 with all but two studies published after the year 2000 [88,89]. A number of articles also have discussed the potential advantages and disadvantages of CI [29,31,46,90,91]. Yet, the limitations of the existing studies have not been analyzed in detail and require further elaboration. Figure 4 briefly summarizes the current limitations associated with the available clinical trials.

**Table 2:** Characteristics of previously published studies for CI vs. IB of beta-lactam antibiotics

**Study**	**Setting (country)**	**Test antibiotic**	**Critically ill?**	**Patient population**	**Sample size**	**Age (yr)**^**a**^		**Allocation sequence generator**	**Allocation concealment**	**Masking**	**Concomitant antibiotics**
						**CI**	**IT**				
Angus et al. [87]	Not specified (Thailand)	Ceftazidime	Yes	Septicemic melioidosis	21	48 (29–58)	43 (27–73)	Not specified	Not specified	Not specified	Amx/clv or doxy, tmp/smx and chlora
Bodey et al. [89]	Non-ICU (USA)	Cefamandole	No	Malignant diseases with neutropenia	204	Not specified		Adequate	Adequate	Not specified	Carbenicillin
Buck et al. [81]	Non-ICU (Germany)	Pip/tazo	No	Hospitalized infections	24	60-88^b^	32-76^b^	Not specified	Adequate	No	Nil stated
Buijk et al. [52]	ICU (Netherlands)	Ceftazidime	Yes	Intra-abdominal infections	18	12 (46–76)	6 (42–87)	Not specified	Not specified	No	Various
Georges et al. [80]	ICU (France)	Cefepime	Yes	Critically ill with gram-negative infections	50	50 ± 17	46 ± 24	Not specified	Not specified	No	Amikacin
Hanes et al. [86]	ICU (USA)	Ceftazidime	Yes	Critically ill trauma	32	33.5 ± 12.5	36.1 ± 12.8	Not specified	Not specified	No	Nil stated
Kojika et al. [82]	Not specified (Japan)	Meropenem	No	Intra-abdominal infections	10	67.4 ± 14.6	60 ± 12.8	Not specified	Not specified	No	Nil stated
Lagast et al. [88]	Not specified (Belgium)	Cefoperazone	No	Gram-negative septicaemia	45	37-77^b^		Not specified	Not specified	No	Nil stated
Lau et al. [79]	ICU (USA)	Pip/tazo	No	Complicated intra-abdominal infections	262	50.4 ± 16.6	49.3 ± 17.8	Not specified	Not specified	No	Nil stated
Lubasch et al. [83]	Not specified (Germany)	Ceftazidime	No	Hospitalized patients with COPD exacerbation	81	65.3 ± 10.1		Not specified	Not specified	No	Nil stated
Nicolau et al. [84]	ICU (USA)	Ceftazidime	Yes	Critically ill patients with sepsis	41	46 ± 16	56 ± 20	Adequate	Not specified	No	Tobramycin
Pedeboscq et al. [85]	ICU (France)	Pip/tazo	Yes	Severe sepsis	7	58 ± 12		Not specified	Not specified	No	Ofloxacin
Rafati et al. [78]	ICU (Iran)	Piperacillin	Yes	Critically ill patients with sepsis	40	50.1 ± 22.2	48.0 ± 20.7	Not specified	Not specified	No	Amikacin
Roberts et al. [75]	ICU (Australia)	Ceftriaxone	Yes	Critically ill patients with sepsis	57	43 ± 19	52 ± 16	Adequate	Adequate	Adequate^c^	Multiple depending on indication
Sakka et al. [77]	ICU (Germany)	Imi/cila	Yes	Critically ill patients with sepsis	20	62 ± 16	59 ± 16	Not specified	Adequate	No	Nil stated
Van Zanten et al. [76]	Not specified (Netherlands)	Cefotaxime	No	Hospitalized patients with COPD exacerbation	93	65.3 ± 8.4	68.6 ± 5.3	Not specified	Not specified	No	Nil stated

CI, continuous infusion; IB, intermittent bolus; Amx/clv, amoxicillin/clavulanic acid; doxy, doxycycline; tmp/smx, trimethoprim/sulphamethoxazole; chlora, chloramphenicol; pip/tazo, piperacillin/tazobactam; Imi/cila, imipenem/cilastatin; COPD, chronic obstructive pulmonary disease.

^a^Values are described as published results as mean (±SD) or median (range).

^b^Values are reported as range.

^c^Only outcome assessment.

### Heterogeneous patient populations

Most of the relevant trials recruited hospitalized patients from different populations, especially the noncritically ill patient groups (Table 2). The diverse range of patient groups include critically ill patients with sepsis [75,78,80,84,85,87,88], trauma patients [86], patients with abdominal infections [79,82], chronic obstructive pulmonary disease (COPD) patients [76,83], cancer [89], and nonspecific hospitalized infections [81]. Thus, meta-analyses have evaluated heterogenous patient groups and any potential benefits of CI or IB that may exist in a particular patient group were not assessed. This issue was previously discussed by Roberts et al. in their meta-analysis, where large confidence intervals were observed suggesting clinical differences may exist between CI and IB administration if more stringent and rigorous inclusion criteria were used in clinical studies [39].

### Inclusion of patients with a low level of illness severity

Detecting significant difference between CI and IB is difficult because the potential benefits may be masked by the inclusion of low-risk patients that have much lower mortality rates than reported in epidemiological studies. This selection bias was further described in two recent meta-analyses [38,39]. For example, two of the nine ICU studies that were meta-analyzed by Roberts et al. reported a mortality rate <10% when the mortality rate for severe sepsis is usually reported between 40% and 50% [92-94] (Table 3). Other studies also recruited patients with low illness severity whereby more than 70% of the cohort presented with an Acute Physiological and Chronic Health Evaluation II (APACHE II) score of only 10 [79]. This is a problem because any differences between CI and IB are more likely emerge in more severely ill patients [45,87]. Critically ill patients with severe sepsis are more likely to benefit from CI, because they commonly develop severe pathophysiological changes, which may reduce effective antibiotic exposure [95]. Furthermore, these patients are usually infected with pathogens that are less susceptible to antibiotic therapy. Thus, in combination, the two important factors may reduce PK/PD target attainment in severely ill patients. In contrast, it has been shown that IB dosing of beta-lactam antibiotics achieves adequate PK/PD target for bacterial eradication in patients with low level of illness severity.

**Table 3:** Antibiotic dosage and outcome data of previously published for CI vs. IB of beta-lactam antibiotics

**Study**	**Types of infection**	**Number of patients (APACHE score**^**a**^**)**		**Antibiotic dosage regimen**		**Concurrent PK/PD analysis**	**Clinical outcome measures**	**CI**	**IB**	***p*value**
		**CI**	**IB**	**CI**	**IB**					
Angus et al. [87]	Melioidosis	10 (15)	11 (21)	12 mg/kg LD, then 4 mg/kg every 1 hr	40 mg/kg every 8 hr	Yes	Mortality	20%	36.4%	0.89
Bodey et al. [89]	Pneumonia, UTI, septicemia and neutropenic fever	167 (ND)	162 (ND)	3 g LD, then 12 g/24 hr	3 g every 6 hr	No	Clinical cure	64.8%	56.5%	ND
Buck et al. [81]	Pneumonia, cholangitis and FUO	12 (ND)	12 (ND)	2 g LD then 8 g/24 hr^b^	4 g every 8 hr^b^	Yes	Clinical response	67%	67%	ND
Buijk et al. [52]	Intra-abdominal infections	12 (16)	6 (14)	1 g LD then 4.5 g/24 hr	1.5 g every 8 hr	Yes	Mortality	25%	33%	1.0
Georges et al. [80]	Pneumonia and septicemia	24 (45^c^)	23 (44^c^)	2 g/12 hrs twice daily	2 g every 12 hr	Yes	Mortality	12%	13%	ND^d^
							Clinical cure	85%	67%	ND^d^
							Duration of MV	24 ± 13	25.3 ± 10	ND^d^
							LOS ICU	34 ± 17	40 ± 15	ND^d^
Hanes et al. [86]	Pneumonia	17 (13)	14 (11)	2 g LD then 60 mg/kg every 24 hr	2 g every 8 hr	Yes	Duration of MV	22.9 ± 19.9	13.3 ± 6.1	0.16
							LOS ICU	26.8 ± 20.1	15.5 ± 5.9	0.11
							LOS Hospital	41.7 ± 30.5	28.7 ± 15.9	0.37
							Duration of leukocytosis	7.8 ± 7.3	11.3 ± 4.7	0.35
							Duration of pyrexia	7.9 ± 4.4	4.3 ± 2.5	0.06
Kojika et al. [82]	Abdominal abscess	5 (ND)	5 (ND)	0.5 g every 8 hr (over 3 hr)	0.5 g every 8 hr (over 30 min)	No	Mortality	20%	0%	
Lagast et al. [88]	Septicemia	20 (ND)	25 (ND)	Day 1: 1 g LD then 3 g/24 hr Day 2 +: 4 g/24 hr	2 g every 12 hr	No	Mortality	25%	16%	ND
							Clinical cure	70%	80%	ND
Lau et al. [79]	Abdominal infections	81 (8)	86 (8)	2 g LD then 12 g/24 hr^e^	3 g every 6 hr^e^	No	Mortality	0.76%	2.6%	
							Clinical cure	86.4%	88.4%	0.817
							Adverse events	89.2%	87.1%	
Lubasch et al. [83]	Chronic bronchitis	41 (ND)	40 (ND)	2 g LD then 2 g/7 hr twice daily	2 g every 8 hr	Yes	Clinical cure	90.2%	90%	ND^d^
							Bacteriological cure	90.2%	87.5%	ND^d^
Nicolau et al. [84]	Pneumonia	17 (14)	18 (16)	1 g LD then 3 g/24 hr^f^	2 g every 8 hr^f^	No	Clinical cure	41%	33%	0.592
							Duration of MV	7.9 ± 4.0	8.3 ± 4.3	0.97
							LOS ICU	8.5 ± 3.4	9.3 ± 4.0	0.691
							Time to defervescence	3.1 ± 2.1	5.2 ± 2.3	0.015
							WBC normalization	7.3 ± 3.0^g^	5.5 ± 4.2^g^	0.259
Pedeboscq et al. [85]	Gastrointestinal-related infections	3 (ND)	4 (ND)	12 g/24 hr	4 g every 8 hr	Yes	Mortality	0%	0%	ND
Rafati et al. [78]	Pneumonia, UTI, abdominal infections, SSI and septicemia	20 (16)	20 (14)	2 g LD then 8 g/24 hr	3 g every 6 hr	Yes	Mortality	30%	25%	0.72
							Decrease in illness severity			CI > IT^h^
							Duration of pyrexia	2.4 ± 1.5	1.7 ± 0.7	0.08
							WBC normalization	75%	83%	ND
Roberts et al. [75]	Septicemia	29 (19)	28 (16)	0.5 g LD then 2 g/24 hr	Day 1: 2.5 g/24 hr Day 2: 2 g/24 hr	No	Mortality	10%	0%	0.25
							Clinical cure^i^	52%	20%	0.04
							Duration of MV	4.3 ± 4.5	3.4 ± 4.1	0.33
							LOS ICU	10.8 ± 23.2	5.6 ± 6.0	0.29
							LOS hospital	42 ± 6.9	24 ± 2.1	0.34
Sakka et al. [77]	Pneumonia	10 (26)	10 (28)	1 g LD then 2 g/24 hr	1 g every 8 hr	Yes	Mortality	10%	20%	ND
Van Zanten et al. [76]	COPD exacerbations	40 (ND)	43 (ND)	1 g LD then 2 g/24 hr	1 g every 8 hr	Yes	Clinical cure	93%	93%	0.93

APACHE, Acute Physiology and Chronic Health Evaluation score; CI, continuous infusion; IB, intermittent bolus; PK/PD, pharmacokinetic/pharmacodynamic; UTI, urinary tract infection; FUO, fever of unknown origin; SSI, surgical site infection; COPD, chronic obstructive pulmonary disease; ND, not described; LD, loading dose; MV, mechanical ventilation; LOS, length of stay; ICU, intensive care unit; WBC, white blood cells.

^a^Values are reported as mean.

^b^Dose reduction in renal dysfunction-if creatinine clearance 25–60 mL/min (CI group 6.75 g/24 hr; IT group 4.5 g q12hr); if creatinine clearance <25 mL/min (CI group 4.5 g/24 hr; IB group 4.5 g every 24 hr).

^c^Values are SAPS II score.

^d^*P* values for nonsignificance were not reported.

^e^Dose reduction in renal dysfunction-if creatinine clearance 20–40 mL/min (CI group 8 g-1 g/24 hr; IB group 2 g −0.25 g every 6 hr).

^f^Dose reduction in renal dysfunction-if creatinine clearance 31–50 mL/min (CI group 2.5 g/24 hr; IB group 2 g every 12 hr); if creatinine clearance 21–30 mL/min (CI group 2 g/24 hr; IB group 2 g every 24 hr).

^g^x 10^9^/L.

^h^Significant difference in APACHE II scores on days 2, 3, and 4.

^i^*A priori* analysis.

### Inconsistent antibiotic dosing regimen

Most of the studies included in the three meta-analyses utilized higher IB doses than the CI treatment arm, potentially favoring the former [76-79,81,84,86,87] (Table 3). This treatment bias might have skewed the results of these meta-analyses towards the null hypothesis. Another logical conclusion is that a lower dose in the CI group was able to achieve equivalent outcomes to a higher dose in the IB group. A significant difference in clinical outcomes might emerge if the two approaches utilized the same daily dose. This notion has been supported by a meta-analysis, whereby clinical failures were less frequent in the CI group, when separate analyses were performed in trials which used the same total daily dose in the two treatment arms [40]. Furthermore, dosing inconsistency with regards to initial bolus administration of antibiotic was reported in several studies [80,82,85] (Table 3). An initial loading dose was not provided to the CI protocol in these studies. This approach delays attainment of target antibiotic concentrations compared with the IB group. To make the two dosing protocols comparable, an initial and equal loading dose of beta-lactam antibiotic should be provided to both groups.

### Pathogens with low MIC values

The offending pathogens isolated in most of the clinical trials have MIC values that make them highly susceptible. Simulation data suggests that there will be little difference in the achievement of PK/PD targets for IB and CI if the pathogens involved are in the susceptible range. When less susceptible pathogens are present, the true potential benefits of CI may be seen because treatment failures are more likely with IB [27,44,57,87]. This limitation has been highlighted in the two most recent meta-analyses and has been proposed as one of the contributing reasons why clinical differences between CI and IB of beta-lactams have not been found. This notion is supported by one randomized, clinical trial [87] and two retrospective, observational studies [45,96], which were conducted in critically ill patients infected with gram-negative organisms. These studies reported clinical cure and mortality benefits favoring CI for pathogens with high MIC values.

### Concomitant administration of other antibiotics

Another noteworthy limitation is that patients included in the available clinical trials were frequently prescribed concomitant antibiotics that, unrelated to the method of beta-lactam administration, may influence clinical outcome [38,40]. Such additional antibiotics (aminoglycosides and fluoroquinolones) provide adequate antimicrobial coverage for most gram-negative pathogens. Therefore, the exclusive contribution of beta-lactam antibiotics to patient outcomes will be poorly defined in these trials. However, in reality, beta-lactam antibiotics are usually administered in conjunction with an aminoglycosides especially when treating patients with severe infections. It follows that regardless of the beta-lactam administration method, the aminoglycosides or fluoroquinolones may be “protecting” patients from gram-negative pathogens during periods of inadequate beta-lactams concentration. A plausible explanation could be that the method of administering beta-lactams is not that important when additional gram-negative coverage (i.e., aminoglycosides or fluoroquinolones) is used.

### Insufficient sample sizes

The lack of significance in the published results also may be attributed to the consistently small sample sizes that have been used to explore the effect of CI vs. IB. The typical study cohort size has varied from 10 to 531 patients but the majority of these trials studied less than 60 patients [52,75,77,78,80-82,84,86,88] (Table 2). The small sample sizes and heterogenous patient background in the clinical studies contribute to insufficient power to investigate the value of both dosing methods. Further to this, if the population of interest is critically ill patients, a single intervention in this setting in very unlikely to influence mortality and clinical cure [31] and, as a consequence, a much larger sample size is needed to show significance [21,40,80]. For instance, Roberts et al. calculated that a sample size of 560 patients in each dosing protocol would be needed to detect difference in bacteriological outcomes [75]. Considering the difficulties in achieving these numbers in critically ill patients, perhaps it is time for clinicians to fully acknowledge the importance of surrogate endpoints in the setting of a study. Clinical cure and ICU-free days are suitable surrogate endpoints and may be used as primary outcomes in a phase II study of this intervention.

### Other relevant concerns

Apart from the discussion above, there are several other plausible reasons why clinical differences have not been established between CI and IB in previous trials. It is important to note that some patients, especially in the ICU, may have some degree of renal impairment on admission or during their hospital stay [97-99]. Whereas a commonly prescribed beta-lactam dose may not be sufficient in patients with mild or no renal impairment, target antibiotic concentrations are more easily achieved in patients with moderate to advanced renal impairment [100]. Similarly, beta-lactam exposure will still be adequate in patients with significant renal impairment as antibiotic clearance is reduced, regardless of the drug administration method [19]. Finally, one of the strongest bodies of evidence suggesting the superiority of CI has been derived from in vivo or animal models. However, the metabolic pathways and tissue distribution patterns of an antibiotic in animals may differ from humans. In addition, more often than not, these animal models fail to mimic human sepsis [101,102].

Based on the discussion above, clearer insights regarding CI and IB administration in patients receiving beta-lactam antibiotics are emerging. Importantly, the results from previous clinical studies suggest that CI of beta-lactam antibiotics is unlikely to be advantageous for all hospitalized patients but may be important in specific patient cohorts. Thus, in an attempt to elucidate the true benefits, we contend that the patient population most likely to adequately test the putative benefits of continuous administration of beta-lactam antibiotics must involve 1) critically ill patients; 2) patients with higher level of illness severity (i.e., APACHE II score ≥ 15); 3) patients infected with less susceptible microorganisms; and 4) patients with gram-negative infections.

### Methodology concerns and the proposed characteristics of an “ideal” trial

Several reviews have suggested the importance of designing and conducting a methodologically sound clinical trial in the investigation of CI versus IB antibiotic administration benefits in critically ill patients [29,38-40]. These recommendations cannot be overemphasized due to frequent reports of low methodological quality clinical studies. It also is noteworthy that a previous study, which utilized the most rigorous and stringent methods, was the only study to demonstrate a clinical cure advantage in favor of CI administration of beta-lactams [75]. The characteristics of an “ideal,” randomized, clinical trial to compare CI vs. IB administration of beta-lactam antibiotics in critically ill patients are described in Table 4.

**Table 4:** Description of a randomized clinical trial that should be performed to investigate CI vs. IB beta-lactam antibiotics

**Criteria**	**Comments**
Population	Should only include patients with sepsis or severe sepsis
Intervention	Antibiotic dosing regimen should be similar between CI and IB group
	a. A loading dose should be given to continuous infusion group to ensure rapid attainment of target antibiotic concentration
	b. An equal daily antibiotic dose should be given to continuous and bolus administration group
	c. Antibiotic doses should be specified according to the patient’s body weight
	d. Concomitant administration of other non-beta-lactam antibiotics should be allowed
PK/PD analysis	Concurrent PK/PD analysis should be performed to support any findings
	a, Measurements of antibiotic concentrations should be performed as long as contributing sites have necessary infrastructure to ensure apt sampling
	b. PK/PD analysis should evaluate the relative ability of IB to achieve a C_min_ greater than 4 x MIC of the offending pathogen for 40-70% of the dosing interval while for CI, a C_ss_ greater than 4 x MIC
Methods	*Design*
	Preferably multicenter in nature and recruits participants from different regions to improve generalizability of results
	*Patients*
	Define eligibility criteria for participants to be included into trial
	a. Definition of sepsis and severe sepsis should be described in detail
	b. Inclusion and exclusion criteria used should be explained
	*Randomization*
	Detailed explanation on allocation sequence generation development
	Detailed allocation concealment mechanism
	*Blinding/masking*
	Outcome evaluators for the trial should be blinded to participants management
	*Endpoints*
	a. Endpoints selection should include primary (clinical outcome) and secondary (PK/PD; adverse event) endpoints
	b. Data collection on the observed bacterial resistance in the two treatment arms should occur

Most studies did not address the methods for allocation sequence generation and concealment further increasing the chance for selection bias [74,76,78-88] (Table 2). In many studies, masking or blinded assessment of the study endpoints were not adequately addressed causing detection bias [74,76-84,86-89] (Table 2). In addition, most of the available studies have failed to have blinded clinicians to assess the clinical and bacteriological outcomes. Each of these issues increases the possibility of systematic errors in these studies.

Ideally, the antibiotic dosing regimen in each treatment arm (i.e., CI and IB) should be comparable in terms of the provision of an initial loading dose with an equal daily antibiotic dose. Alternatively, a suitable surrogate endpoint, such as a cost-effective analysis, may be used to compare these two dosing approaches when unequal antibiotic doses are used between them. Concomitant administration of other non-beta-lactam antibiotics, such as the aminoglycosides or fluoroquinolones, in the setting of a study is appropriate considering that a single empirical therapy is unlikely to occur during antibiotic initiation in ICU patients [1,4]. Furthermore, the concomitant administration of other antibiotics should be regarded as a limitation rather than a major flaw in future trials, because it reflects real clinical practice. However, the number of concomitant antibiotics that are used and their administration sequences in relation to the main study antibiotic should at least be described in the trials.

Only a number of studies measured the antibiotic concentrations and included a concurrent PK/PD evaluation to confirm whether the dosing approaches were actually meeting their respective PK/PD endpoints (Table 3) [52,76-79,86]. Hence, it is difficult to relate clinical outcomes to the respective antibiotic exposure obtained via the two approaches. It is imperative to note the difference in PK/PD endpoints with regards to CI and IB. In a comparison that reflects current practice, CI has to achieve a steady-state antibiotic concentration (C_ss_) greater than 4 x MIC to be microbiologically more effective than IB. On the other hand, IB has to achieve an antibiotic concentration (C_min_) greater than 4 x MIC during the typical 40–70% of the dosing interval [29,33,87,103]. Therefore, concentrations measurement and concurrent PK/PD analysis needs to be done in light of this information or the extent of antibiotic exposure may not occur as predicted and therefore the influence on clinical cure and mortality will never be understood. However, the concurrent analysis should only be considered “compelling” in future trials as long as contributing clinical sites have the necessary infrastructure to ensure apt antibiotic sampling. Errors in sampling, which are common in PK/PD studies, may result in inaccurate and faulty assessments with regards to the apparent benefits of either dosing approaches.

Considering the rampant development of bacterial resistance, the exact role of altered dosing approaches to reduce the problem should be addressed. To date, studies investigating the impact of various beta-lactams dosing approaches and their associated risk of bacterial resistance are scarce [72]. However, it is interesting to note the findings of a recent prospective, multicenter, randomized study that compared the clinical benefits of EI doripenem vs. IB imipenem in patients with ventilator-associated pneumonia [74]. The authors reported that only 18% of patients treated with EI developed resistance of *P. aeruginosa* compared with 50% who received the conventional imipenem dosing. This is one of the few recent studies to evaluate the relative ability of the two dosing approaches in the prevention of resistance; similar studies, particularly involving critically ill patients, should ensue. Although there are not enough prior clinical data to power a study in the ICU and describe the appropriate methodology, data collection on the observed resistance should be performed in future clinical trials investigating the two dosing approaches.

Previously published studies have mostly been single-center in design. To our knowledge, only three studies were conducted as a multicenter study, and only two of the three studies were able to include more than 200 patients [74,79,83]. The need for more multicenter studies should be emphasized, because these studies will provide a stronger basis for subsequent generalization of any findings. Participation from different regions and countries in such studies also should be encouraged to facilitate generalization even more to an extent of a possible global practice change.

Because of the cost of large-scale trials, a step-wise approach to consider potential problems and feasibility is desirable. An initial pilot study before proceeding with a larger multicenter trial is beneficial. In this regard, the Beta-lactam Infusion Group’s feasibility study (BLING I) has now led to the design of large clinical outcome study, BLING II. BLING I (Dulhunty et al., unpublished results; ACTRN12610000238077) was a prospective, multicenter, double-blind, double dummy, pilot, randomized, controlled trial enrolling 60 critically ill patients from 5 ICUs across Australia and Hong Kong. The primary endpoint of the study was to establish the PK separation between CI and IB in terms of achieving plasma antibiotic concentrations above the MIC of causative pathogens. The PK findings in BLING I demonstrated significant differences in plasma antibiotic concentrations above MIC favoring the CI group (CI; 81.8% vs. IB; 28.6%, *p* = 0.001), thus supporting the notion of PK/PD superiority associated with continuous administration. Clinical cure also was superior in the CI group (CI; 70.0% vs. IB; 43.3%, *p* = 0.037). Other relevant findings include the feasibility of the proposed randomization and blinding process used by the BLING I investigators and the suggestion of appropriate surrogate endpoints for survival to be utilized in a multicenter study. Thus, based on the findings from BLING I, BLING II was designed with rigorous and stringent methods to answer the ultimate question of whether administration of beta-lactam antibiotics by CI will result in improved outcomes for patients with severe sepsis. BLING II is a phase II, multicenter, double-blinded, randomized, controlled trial that will recruit critically ill patients with severe sepsis in several ICUs in New Zealand as well as Australia and Hong Kong. The Australian National Health and Medical Research Council (NHMRC)-funded clinical trial aims to compare the effects of two approaches to the administration of beta-lactam antibiotics (i.e., CI vs. IB) on ICU-free days up to day 28.

## Conclusions

Although numerous PK/PD data from various in vitro and in vivo experimental studies favor the use of continuous infusion, the current clinical data are less convincing and insufficient to instigate a global shift from conventional bolus dosing. However, this lack of convincing data may be due to several methodological flaws and inconsistencies among the available studies, thus contributing toward insufficient power to detect any significant differences between CI and IB, if they exist. Based on the published literature, it can be concluded that CI of beta-lactams will not be beneficial to all patients but may potentially be beneficial to specific subsets of patients. If any patient group is likely to benefit from CI, it may be critically ill patients with severe infections. If benefits from CI do exist in critically ill patients, a large-scale, prospective, multinational trial with a robust design is required. A step-wise approach to conduct such clinical trials has begun and already shows promise. A phase II study involving 420 patients is about to start and will provide high-quality information to confirm or refute the need for a pivotal phase III double-blinded RCT of CI vs. beta-lactam dosing in critically ill patients with sepsis.

## Abbreviations

PK, Pharmacokinetics; PD, Pharmacodynamics; ICU, Intensive care unit; MIC, Minimal inhibitory concentration; Cmax, Maximal concentration; AUC0-24, Area under the concentration time curve during a 24-hour period; AUC0-24/MIC, Ratio of area under the concentration time curve during a 24-hour period; PAE, Post antibiotic effect; CI, Continuous infusion; IB, Intermittent bolus; EI, Extended infusion; Cmin, Lowest concentration; APACHE II, Acute Physiological and Chronic Health Evaluation II; T, Time; Css, Steady-state concentration; BLING, Beta-lactam Infusion Group; NHMRC, Australian National Health and Medical Research Council; RCT, Randomized controlled trial; ISF, Interstitial fluid.

## Competing interests

Mohd Abdul Aziz, Dr. Joel Dulhunty, and Prof Rinaldo Bellomo have no conflicts of interest. Dr. Jason Roberts has served for a consultant for Astra Zeneca, Pfizer, Gilead, and Janssen-Cilag. Prof. Jeffrey Lipman has received research grants from Astra Zeneca and has attended Advisory Boards, acted as a consultant to, or given lectures with honoraria from Astra-Zeneca, Janssen-Cilag, Merck Sharp & Dohme, Pfizer, and Wyeth Australia.

## Authors’ contributions

JD and RB conceived the topic review idea and proposal. MA-A, JD, JL, and JR performed the literature search and selected the relevant articles to be discussed. All authors participated in design, coordination, and writing of the manuscript. All authors read and approved the final manuscript.
